# Employment profiles of autistic people: An 8-year longitudinal study

**DOI:** 10.1177/13623613231225798

**Published:** 2024-01-19

**Authors:** Simon M Bury, Darren Hedley, Mirko Uljarević, Xia Li, Mark A Stokes, Sander Begeer

**Affiliations:** 1 La Trobe University, Australia; 2 The University of Melbourne, Australia; 3 Stanford University, USA; 4 Deakin University, Australia; 5 Vrije Universiteit Amsterdam, The Netherlands

**Keywords:** autism, employment, longitudinal, person-oriented methods, trajectory analysis, unemployment

## Abstract

Autistic adults experience high rates of unemployment, yet research investigating what predicts employment success produces inconsistent results. By utilising longitudinal person-oriented analyses, this study aimed to identify employment trajectories of autistic adults to better understand what may predict stable autistic employment. Participants were 2449 autistic adults (1077 men, 1352 women, 20 non-binary, M *=* 42.25 years, SD = 14.24), recruited via the Netherlands Autism Register. Latent class analysis utilising employment status across eight annual waves was used to identify longitudinal employment profiles. Fit indices and the interpretability of results indicated a four-class model best fit the data, with profiles reflecting stable unemployment (*n* = 1189), stable employment (*n* = 801), early unemployment increasing in probability of employment (*n* = 183) and high probability of employment reducing across time to low employment (*n* = 134). Multinominal analyses suggested that compared to the ‘stable unemployment’ group, membership in the ‘stable employment’ profile was predicted by fewer autistic traits, lower age, male gender, higher education and diagnosis age, and fewer co-occurring conditions. Higher education predicted both other profiles, with lower age and fewer co-occurring conditions predicting membership in the increasing employment class. Taken together, findings highlight the utility of person-oriented approaches in understanding the longitudinal challenges autistic adults experience maintaining employment and identifies key areas of support.

**Lay abstract:**

Autistic adults experience difficulties finding and keeping employment. However, research investigating reasons that might explain this difficulty produce mixed results. We gave a survey to 2449 autistic adults and used a statistic method to group them based on their employment status over 8 years. We identified four employment groups that best captured the experiences of autistic adults; this included a group that experienced stable unemployment, a group that experienced stable employment, a group that had high employment that reduced over time, and a group whose employment increased over the 8 years. Further analysis showed that those with fewer autistic traits, younger age, male gender, higher education, later diagnosis age and no co-occurring conditions were more likely to have stable employment. People whose employment changed over time were more likely to have a higher level of education than the stable unemployment group, and those in the increasing employment group were younger age and had no co-occurring conditions. These findings help us better understand that not all autistic adults’ experiences of employment are the same, which helps focus where employment programmes and support may be most needed, for example, people who identify as women or have a co-occurring condition.

Autistic people are significantly underrepresented in employment, with low employment rates across the globe (e.g. Australia, 27.3%; Israel, 28%; the United Kingdom, 29%; the United States and Canada, 14%; Australian Bureau of Statistics, 2019; Beenstock et al., 2020; Office for National Statistics, 2022; Roux et al., 2017; Zwicker et al., 2017). In Australia, this is lower than adults with no disability (80.3%), and all other disability groups (47.8%; Australian Bureau of Statistics, 2019), with similar disparities between autism and other disabilities (e.g. intellectual disability) in the United States (Roux et al., 2017). Even when employed, autistic people face underemployment and lower pay (Cimera & Cowan, 2009). Although there is strong evidence of heterogeneity in population study findings (Steinhausen et al., 2016), when considering global outcomes for autistic adults, only around one quarter of autistic individuals achieve what can be considered good occupational and social outcomes (Howlin, 2013; Howlin et al., 2004; Howlin & Magiati, 2017). While employment is not always the aim or appropriate for all autistic people, many autistic people report a strong desire to work (Chen et al., 2015). Improving employment outcomes has the potential to improve quality of life, social participation, and well-being of autistic individuals and their families (Hedley et al., 2019), and with 36% of costs supporting autistic adults attributed to lost employment (Buescher et al., 2014), increasing access to employment significantly reduces the economic impact for individuals and society (Hedley et al., 2023; Leigh & Du, 2015; Mavranezouli et al., 2014).

Although there is much to be gained from comprehensive understanding of factors associated with specific employment outcomes in autistic people, previous research investigating predictors of autistic employment has utilised mostly variable-oriented approaches, which focus on predictor variables while assuming the sample is a homogeneous group. Such approaches have produced largely inconsistent findings regarding specific predictors of autism employment. To address noted limitations, in this study, we adopted a person-oriented approach to identify longitudinal employment profiles of autistic adults. More specifically, we aimed to (1) better account for the heterogeneity in autism presentation and findings by identifying employment profiles of autistic individuals utilising longitudinal employment success trajectories and (2) characterise factors associated with individuals’ alignment with employment profiles.

In the following sections, we provide a review of factors associated with employment success based on variable-oriented approaches, before making the case for a longitudinal person-oriented approach.

## Predictors of employment

Although previous cross-sectional (e.g. Harvery et al., 2021; Maslahati et al., 2022; Ohl et al., 2017) and longitudinal research (e.g. Beenstock et al., 2020; Chan et al., 2018; Chiang et al., 2013; Wong et al., 2021) have explored a broad and diverse set of potential predictors of employment, they have yielded mostly inconsistent findings. A summary of findings follows with predictors classified into broader individual, family and vocation categories.

### Individual factors

Individual difference factors suggest that autistic males have greater success in employment (e.g. Alverson & Yamamoto, 2017; Holwerda et al., 2012; Taylor & Mailick, 2014), although females have the advantage in some studies (Chiang et al., 2013), or gender is a non-significant predictor in others (e.g. Kaya et al., 2016; Maslahati et al., 2022; Ohl et al., 2017). Ethnicity has been associated with employment success, with primarily White or dominant cultures associated with better employment outcomes (Alverson & Yamamoto, 2017; Kaya et al., 2016), although ethnicity is not always a significant contributor (Migliore et al., 2012). Age has not been significant predictor of gaining employment (Migliore et al., 2012; Taylor & Mailick, 2014). Education level, however, is a consistent predictor of employment (Alverson & Yamamoto, 2017; Chiang et al., 2013; Ohl et al., 2017).

For factors associated with autism, later age of diagnosis has been associated with better employment outcomes (Beenstock et al., 2020). Autism traits as measured by standard measures of broad autism traits (e.g. restrictive behaviours, social differences) have been traditionally linked to worse adult outcomes (Holwerda et al., 2012; Howlin & Magiati, 2017), with increased autism traits as measured by the Autism Diagnostic Interview–Revised (ADI-R; Lord et al., 1994) a negative predictor of employment in a broad sample (Taylor et al., 2015; Taylor & Seltzer, 2011); however, increased autism traits as measured by the Autism Spectrum Quotient–Short (AQ-Short; Hoekstra et al., 2011) were associated with better employment outcomes in a small Australian study (Harvery et al., 2021). Other research shows co-occurring conditions (e.g. psychiatric disorder, epilepsy) negatively predicting employment outcomes (Holwerda et al., 2012), although not in all studies (Ohl et al., 2017).

Cognitive functioning either as the presence of an intellectual disability (Taylor & Mailick, 2014; Taylor & Seltzer, 2011) or lower scores on intelligence tests (Holwerda et al., 2012) have been consistently negatively related to employment (Maslahati et al., 2022). More adaptive behaviour or daily living skills have been consistently associated with improved employment outcomes (Beenstock et al., 2020; Shattuck et al., 2012; Taylor & Mailick, 2014), including for participants with co-occurring intellectual disability (Chan et al., 2018). Behaviours of concern that can impede inclusion are consistently associated with worse employment outcomes (Holwerda et al., 2012; Taylor et al., 2015; Taylor & Mailick, 2014). Previous systematic reviews have shown challenges with social interaction impacting employment outcomes (Holwerda et al., 2012), with more support in a recent study (Chiang et al., 2013); however, other studies have not found significant associations (Beenstock et al., 2020; Chan et al., 2018).

### Family factors

Family factors may also impact the employment success of autistic people. Parental level of education (Chiang et al., 2013), their attitudes about the importance of employment (Holwerda et al., 2013), or their efforts to assist their children find work or hiring them directly (Holwerda et al., 2012) have been associated with gaining access to employment. For children with a co-occurring intellectual disability, the size of the mother’s social network was associated with better outcomes (Chan et al., 2018); however, parental support network more broadly was not a significant predictor in a sample of autistic adults with and without intellectual disability (Taylor & Mailick, 2014). Household income has commonly been associated with employment success (Chan et al., 2018; Chiang et al., 2013; Shattuck et al., 2012); however, in another study, this association was significant only for the father’s income (Beenstock et al., 2020), and parental income was non-significant in another (Taylor & Mailick, 2014).

### Vocational factors

Formal programmes that aim to prepare autistic people for work through guidance and coaching have been shown to successfully support autistic people in finding work, such as vocational rehabilitation/training (primarily in the United States), school interventions, targeted programmes (Hedley et al., 2023) or job counselling (e.g. Alverson & Yamamoto, 2017; Chiang et al., 2013; Kaya et al., 2016). While job search assistance is a common predictor across some studies (Kaya et al., 2016; Migliore et al., 2012), what contributes to success can differ. For example, vocational rehabilitation counselling and on-the-job support were significant predictors in one study (Kaya et al., 2016), but not in another (Migliore et al., 2012). Disclosure of autism diagnosis to the employer was a strong predictor of employment success (Ohl et al., 2017). It has been suggested that disclosure is an important factor because it starts a conversation for workplace supports, with such supports (e.g. workplace social supports or adjustments) indicative of employment success for participants in Australia (Flower et al., 2019; Harvery et al., 2021; Hedley et al., 2023).

## Longitudinal person-oriented analyses

Given the significant heterogeneity within autism and pronounced variability in long-term outcomes (Howlin & Magiati, 2017; Steinhausen et al., 2016), variable-oriented analyses, which treat autism as a unitary group, may impede our ability to identify predictors of different employment outcomes. Furthermore, given the instability of autism employment (Baldwin et al., 2014; Hendricks, 2010), employment success might be better conceptualised by changes in employment over time rather than as a singular outcome.

Rather than variable-oriented analyses, other studies investigating long-term employment outcomes of autistic adults have used person-oriented approaches. Person-oriented approaches are data-driven approaches, which aim to approximate real-life trajectories or profiles of participants from the data. In employment, this approach has been used to identify profiles of employment success. However, studies using this approach are few and underpowered. For example, in a small subsample of autistic adults with intelligence quotient (IQ) in normative range (*n* = 73) from a broader study (*n* = 406), Taylor et al. (2015) found with four annual time points that only 24.7% of participants were consistently engaged in employment, 42.5% sometimes engaged in employment and 32.9% never having employment. Factors such as gender, parental education, maladaptive behaviours or autism symptoms were predictive of those who consistently engaged with employment. Utilising a population-based nationwide register in Sweden across four time points, Lallukka et al. (2020) used group-based trajectory analyses (GBTA) with a sample of non-autistic participants (*n* = 22,013) to derive trajectory profiles based on unemployment status. Results suggested three distinct trajectories: initially low and then sharply increasing unemployment (9%), stable low unemployment (67%), and initially high and then slowly decreasing unemployment (24%). When these profiles were applied to the autistic population in the sample (*n* *=* 814, age 19–35 years), having an autism diagnosis was associated with a higher likelihood of being in the first and last trajectory.

However, as discussed above, autistic adults of working age are more likely than non-autistic people to change jobs more frequently (and earn less) than peers with equivalent qualifications, training and experience (Baldwin et al., 2014; Hendricks, 2010), so there may be greater variability in autistic employment profiles than described previously (i.e. Lallukka et al., 2020). When Lallukka et al. (2020) ran the GBTA with the autistic population, they only found one cubic shaped trajectory reflecting vast changes in employment status in the lower range; however, they suggested that potentially more distinct trajectories would emerge given greater statistical power. Sociodemographic factors such as education, being male, age and level of urbanicity (small vs large city) were all associated with likelihood of belonging to the unstable employment groups in this study.

## Current study

To better characterise the constellation of factors driving the longitudinal changes in employment status over time, we used latent class analysis (LCA) to establish distinct profiles of autistic employees based on their employment status across eight time points. Given that most studies thus far have been underpowered, this project capitalised on an extensive, well-characterised annual online survey of over 2400 autistic adults, parents and legal representatives, the Netherlands Autism Register (NAR), to establish unique longitudinal employment profiles over an 8-year period. As LCA is an exploratory technique, we first identified potential profiles using fit measurements and then reviewed these to identify categories that were conceptually meaningful. Based on the studies reviewed above, we predicted there would be a small group of successfully employed individuals, a larger group who were generally unsuccessful in employment, and further profiles that reflect more episodic or sporadic employment profiles (Baldwin et al., 2014; Hendricks, 2010; Lallukka et al., 2020; Taylor et al., 2015).

To further characterise individuals who represent specific employment profiles, we used multinominal analyses with individual, family and vocational factors from the established literature to understand what factors were associated with membership in a specific profile. Given the inconsistent findings of what factors predict employment in variable-oriented approaches, it is important to consider factors anew in this new analytical context. Therefore, we have chosen variables across the literature, including predictive factors in the recent person-oriented studies, that were also available in the NAR. Given the exploratory nature of LCA, making specific predictions is not possible; however, we expected individual factors more commonly associated with employment success (e.g. autism traits, gender, co-occurring conditions, treatment of co-occurring conditions, urbanicity) would be associated with profiles that reflect reduced employment success. Similarly, family factors, such as parental income and importance of employment, and vocational factors, such as workplace guidance and counselling, would be associated with profiles reflecting greater success in employment.

## Method

### Participants

Participants were 2449 autistic adults (1077 men, 1352 women, 20 non-binary), with a mean age of 42.25 years (SD = 14.24) at their most recent wave and reported a later age of diagnosis (M = 33.44 years, SD = 15.93). Participants were recruited through the NAR. Data represented baseline (Wave 0) and seven annual waves collected between 2013 and 2021 (Waves 1–7). To be included in the study, participants had to report an autism diagnosis and be aged at least 16 years of age. Confirmation of an autism diagnosis was established by an authorised professional (e.g. psychiatrist) upon registration into the NAR. While parents and legal representatives are included in the NAR, this study only includes self-report data from autistic adults.

### Procedure

The variables analysed in the current study were collected through the NAR, which is a longitudinal dataset administered on a yearly basis to individuals with an autism diagnosis by an independent qualified clinician (e.g. psychiatrist) in a professional setting (e.g. mental healthcare clinic). For each data wave, existing participants are invited to complete the current version of the study, with new participants recruited at the same time. Researchers seeking to use the NAR complete a data request identifying variables of interest and hypotheses, and if approved, must preregister their research plan prior to receiving the data. This study was registered with Open Science Foundation (Hedley et al., 2020). The research has been evaluated and approved by the ethics committee of the Vrije Universiteit Amsterdam (VCWE 2015/2021-041R1).

### Measures

Predictors were chosen that reflect common predictors of employment in the broader literature, including individual, family and vocational factors.^1^

#### Autism traits

The Abridged Version of the Autism Spectrum Quotient (Hoekstra et al., 2011) is a 28-item self-report measure, measuring the extent of autism traits (e.g. social skills, routine) in individuals. Participants responded to statements on a 4-Point Likert-type scale (1 = definitely agree, 4 = definitely disagree; range = 28–112), with higher scores indicating higher levels of total autism traits. The McDonald’s Omega value of the current sample indicated internal reliability was high (ω *=* 0.837).

#### Employment status

Employment (0 = unemployed, 1 = employed) was calculated for participants who reported participating in competitive employment (Regular paid work; Self-employed) for at least 1 h a week on a measure of daily activities (for more information, see Bury et al., 2024).

#### Gender

To ensure adequate power for analyses, we excluded participants who indicated a non-binary gender.

#### Co-occurring conditions

Participants indicated whether they had a co-occurring diagnosis in addition to autism, and participants who indicated they did not know were recoded as ‘missing’ for analyses.

#### Participant highest level of education

Autistic participants were asked to choose from 20 education options which were recoded into Low (special primary and secondary education), Medium (primary and secondary school) and High (vocational and university). Due to the small number of participants in the low education group, we combined the low and medium levels of education into a single category.

#### Parental highest level of education

Both fathers and mothers separately chose from 14 education options which were recoded into Low, Medium and High levels of education. Father and mother data were combined, with the highest level of education of either parent reported for the current study. Similar steps to the participant level of education were used to establish binary high and low education.

#### Urbanicity

Urbanicity reflects the size of residential area to provide information on opportunity for employment and was measured on a 5-point scale (1 = Very highly urbanised municipality; 2 = Highly urbanised municipality; 3 = Moderately urbanised municipality; 4 = Slightly urbanised municipality; 5 = Non-urban municipality).

### Analysis plan

LCA is a statistical technique used to identify unobserved (latent) subgroups or classes within a population based on observed categorical or discrete variables (Weller et al., 2020). It is a type of finite mixture modelling approach that assumes the population consists of distinct groups, each characterised by a unique pattern of responses. LCA was used in this project to classify participants into subtypes of employment status. The final model was selected by considering the combination of the following fit indices: (1) the Akaike information criterion (AIC); (2) the Bayesian information criterion (BIC); (3) the sample-size adjusted BIC (aBIC); (4) the bootstrap likelihood ratio test (BLRT); (5) Vuong–Lo–Mendell–Rubin likelihood ratio test (VLMR); and (6) entropy values (Morin et al., 2016; Nylund-Gibson & Choi, 2018). A better absolute fit was indicated by lower AIC, BIC, and aBIC values and higher entropy. The VLMR and BLRT are relative fit indices that assess fit improvement with the addition of each subgroup (Nylund-Gibson & Choi, 2018). In addition, model selection was also guided by parsimony and interpretability. Although no prior studies have attempted to utilise LCA approach, based on the previous studies examining both different trajectories and to ensure that any relevant profiles were captured, models with up to six profiles were estimated.

Once latent classes were identified, we employed multinomial analysis to characterise associations between the latent classes and other variables, such as family and vocational factors.

### Community involvement

Autistic individuals are employed in the NAR team. Autistic adults are also consulted through panels. This consultancy includes checking the content, communication (introduction of topics) and explanations of individual feedback. Moreover, the work is consistent with the Dutch Autism Research Agenda, developed by autistic researchers using NAR data (Van den Bosch & Weve, 2019).

## Results

### Employment profiles

Using LCA, we identified models with two to six classes of employment status. The model fit indices are summarised in Table 1. A four-class model was chosen that showed the best fit as measured by a lower BIC as well as the most interpretable and robust individual classes/subgroups (information on the additional class models can be found in the Supplementary file).

**Table 1.** Model-fit indices for the latent lass analysis model.

Number of classes	Log-likelihood	Resid. df	BIC	aBIC	AIC	Likelihood ratio	Entropy
2	−3722.5	238	7576.64	7522.627	7593.64	277.6389	0.791
3	−3654.97	229	7511.275	7428.668	7537.275	206.8948	0.660
**4**	−3557.46	220	**7385.949**	**7274.748**	**7420.949**	136.4807	0.645
5	−3576.3	211	7493.314	7353.518	7537.314	130.2968	0.598
6	−3568.09	202	7546.59	7378.199	7599.59	122.5502	0.582

BIC: Bayesian information criterion; aBIC: adjusted Bayesian information criterion; AIC: Akaike information criterion.

Bold values indicate optimal model based on fit indices (e.g. BIC).

**Figure 1.**
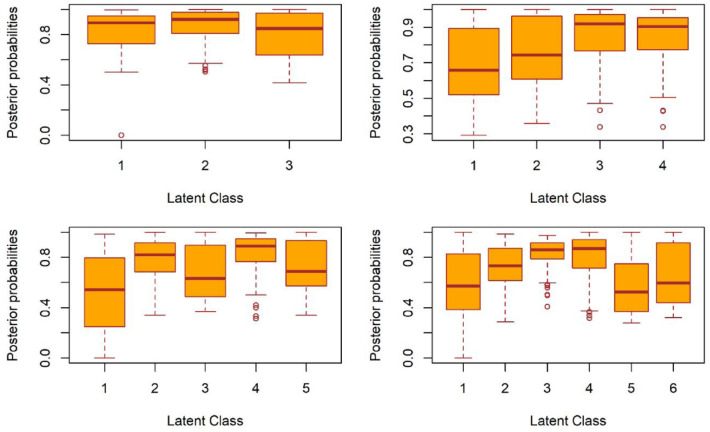
The posterior probabilities distribution for different classes.

Figure 1 shows the posterior probabilities of employed distributions for 3–6 classes. These probabilities represent the likelihood or certainty of an individual’s membership in each class based on their observed responses. Higher probabilities indicate a stronger likelihood of belonging to a specific class, while lower probabilities suggest uncertainty or potential membership in multiple classes. The posterior probabilities for all classes in both the 3-class and 4-class models are consistently above 0.5, which suggests good LCA estimations.

**Figure 2.**
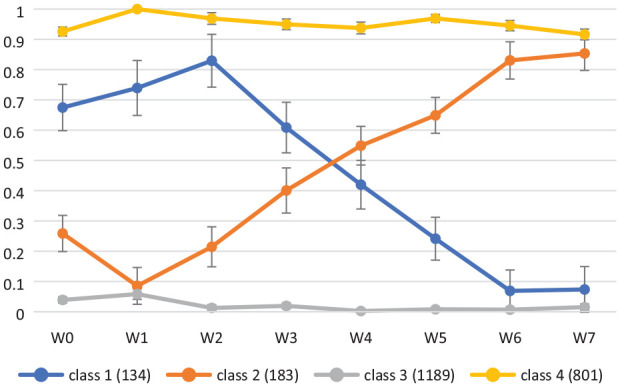
Probabilities of employment in the four latent classes. Class 1: reducing employment; Class 2: increasing employment; Class 3: unemployed; Class 4: employed.

Figure 2 shows probabilities of employment in the four latent classes. Class 3 subjects were the largest class (*n* = 1189) and were characterised by stable unemployment (unemployed class). Class 4 (*n* = 801) kept employment status stable over the study period (employed class). Class 2 subjects generally are those unemployed at the start but show a steady progress of employment (*n* = 183; increasing employment class). Class 1 (*n* = 134) had relatively high probability of employment in baseline (Wave 0), but this reduced to mostly unemployed by the end of the study (reducing employment class). The baseline characteristic of the participants in each of the four classes can be found in Table 2.

**Table 2.** Baseline characteristics according to LCA-derived classes (four classes).

	Overall	1 – Reducing employment	2 – Increasing employment	3 – Unemployed	4 – Employed	*p* value
	(*N* = 2307)	(*N* = 134)	(*N* = 183)	(*N* = 1189)	(*N* = 801)	
AQ						<0.001^a^
Mean (SD)	83.0 (11.3)	83.1 (10.6)	81.3 (10.4)	84.0 (11.7)	82.0 (11.0)	
Missing	327 (14.2%)	6 (4.5%)	13 (7.1%)	242 (20.4%)	66 (8.2%)	
Age						<0.001^a^
Mean (SD)	39.3 (12.6)	40.6 (12.4)	35.4 (12.1)	38.7 (13.4)	40.8 (11.1)	
Gender						<0.001^b^
Male	975 (42.3%)	51 (38.1%)	76 (41.5%)	459 (38.6%)	389 (48.6%)	
Female	1312 (56.9%)	82 (61.2%)	105 (57.4%)	718 (60.4%)	407 (50.8%)	
Excluded^c^	20 (0.9%)	1 (0.7%)	2 (1.1%)	12 (1.0%)	5 (0.6%)	
Education						<0.001^b^
Low	899 (39.0%)	38 (28.4%)	70 (38.3%)	547 (46.0%)	244 (30.5%)	
High	821 (35.6%)	50 (37.3%)	73 (39.9%)	306 (25.7%)	392 (48.9%)	
Missing	587 (25.4%)	46 (34.3%)	40 (21.9%)	336 (28.3%)	165 (20.6%)	
Parent education						0.426^b^
Low	1133 (49.1%)	66 (49.3%)	86 (47.0%)	574 (48.3%)	407 (50.8%)	
High	890 (38.6%)	59 (44.0%)	83 (45.4%)	443 (37.3%)	305 (38.1%)	
Missing	284 (12.3%)	9 (6.7%)	14 (7.7%)	172 (14.5%)	89 (11.1%)	
Age of diagnosis						<0.001^b^
Mean (SD)	33.7 (14.8)	35.9 (13.9)	30.4 (14.1)	32.4 (16.0)	35.9 (12.7)	
Missing	179 (7.8%)	7 (5.2%)	15 (8.2%)	110 (9.3%)	47 (5.9%)	
Co-occurring conditions						<0.001^b^
No	1059 (45.9%)	63 (47.0%)	89 (48.6%)	451 (37.9%)	456 (56.9%)	
Yes	1127 (48.9%)	64 (47.8%)	86 (47.0%)	668 (56.2%)	309 (38.6%)	
Missing	121 (5.2%)	7 (5.2%)	8 (4.4%)	70 (5.9%)	36 (4.5%)	
Urbanicity						0.027^a^
Mean (SD)	2.54 (1.30)	2.37 (1.28)	2.46 (1.29)	2.64 (1.33)	2.43 (1.25)	
Missing	102 (4.4%)	4 (3.0%)	4 (2.2%)	53 (4.5%)	41 (5.1%)	

LCA: latent class analysis; AQ: Autism Spectrum Quotient; SD: standard deviation.

a Kruskal–Wallis rank-sum test.

b Pearson’s chi-square test.

c Non-binary participants were excluded from multinominal analyses to ensure statistical power.

### Multinominal analyses

Multinominal logistic regression was run to investigate what factors predict group membership with the unemployed class as the comparison group (Table 3). Higher levels of education significantly predicted membership in each of the three employment classes compared to the unemployed class, with the odds of participants being a member of a class with some employment as 1.84- to 2.38-fold higher than being in the unemployed class. Parental education level did not predict group membership. The odds of membership in the increasing employment class reduced by 7% with additional increase of 1 year of age and by 48% for those that reported a co-occurring condition. Membership in the employed over the unemployed class was reduced by 6% with each additional increase of 1 year of age and by 2% for each additional score increase in autism traits measured on the AQ-Short. Being a part of the employed class reduced by 57% for participants who reported a co-occurring condition and by 56% for female participants. Membership in the employed class increased by 4% with each year increase in age of diagnosis. Urbanicity did not explain class membership.

**Table 3.** Results of the multinomial logistic regression of predictors of class membership with a 4-class model with the unemployed class as the comparison group.

Profile	1 – Reducing employment			2 – Increasing employment			4 – Employed		
	OR	95% CI	*p*	OR	95% CI	*p*	OR	95% CI	*p*
AQ	1.01	0.99, 1.04	0.3	0.98	0.96, 1.00	0.082	0.98	0.97, 1.00	**0.011**
Age	1	0.94, 1.05	0.9	0.93	0.89, 0.98	**0.003**	0.94	0.92, 0.97	**<0.001**
Gender									
Male	–	–		–	–		–	–	
Female	0.74	0.42, 1.30	0.3	0.64	0.40, 1.03	0.068	0.44	0.33, 0.59	**<0.001**
Education									
Low	–	–		–	–		–	–	
High	1.84	1.08, 3.13	**0.024**	2.38	1.53, 3.70	**<0.001**	2.36	1.80, 3.09	**<0.001**
Parent education									
Low	–	–		–	–		–	–	
High	1.04	0.60, 1.80	0.9	1.04	0.67, 1.62	0.9	1.12	0.84, 1.48	0.4
Dx Age	1	0.96, 1.05	>0.9	1.02	0.98, 1.06	0.4	1.04	1.01, 1.07	**0.002**
Co-occurring									
No	–	–		–	–		–	–	
Yes	0.6	0.35, 1.01	0.053	0.52	0.33, 0.80	**0.003**	0.43	0.33, 0.56	**<0.001**
Urbanicity	0.87	0.71, 1.07	0.2	1.04	0.88, 1.22	0.7	0.94	0.85, 1.05	0.3

OR: odds ratio; CI: confidence interval; AQ: Autism Spectrum Quotient.

Bold equals significant at <0.05.

## Discussion

With low rates of employment reported for autistic people worldwide (Australian Bureau of Statistics, 2019; Beenstock et al., 2020; Office for National Statistics, 2022; Roux et al., 2017), including participants in this sample (~40% employed across waves, Bury et al., 2024), research has sought to understand the employment experience of autistic adults and what factors might help identify barriers to employment. However, most research has treated autistic people as a unitary group and employment outcomes based on a single time point, with findings showing great heterogeneity in employment outcomes, as well as variability in what predicts employment success. To understand this heterogeneity, this current study applied a longitudinal person-oriented approach to one of the largest and most extensively characterised longitudinal cohorts of autistic adults to identify distinct profiles of autistic people based on their employment success over an 8-year period. Overall, we identified four distinct and interpretable longitudinal employment trajectory profiles.

The largest profile represented a group of people characterised by high probability of stable unemployment. Given the overall high rates of unemployment in the autistic population (e.g. Roux et al., 2017; Zwicker et al., 2017), including this sample (Bury et al., 2024), it is not surprising that this was the largest group. The next largest group represented a class typified by high probability of stable employment over time. Finally, two groups reflected mirrored trajectories of high probability of unemployment transitioning gradually to higher likelihood of employment, and the converse, relative early success in employment gradually decreasing to higher probability of unemployment. The latter three groups largely reflect the three groups identified in the general population of Sweden (Lallukka et al., 2020); we have built on these findings in a population of autistic people from the Netherlands, with the unemployed class unique to an autistic population. Importantly, class membership was differentiated by individual factors included in the study.

The participant’s highest level of educational attainment was the most consistent indicator differentiating individuals with some employment from the unemployed class. However, the likelihood of being in the employed class and the increasing employment class were higher than reducing employment class. Consistent with research in other autism studies (Alverson & Yamamoto, 2017; Chiang et al., 2013; Ohl et al., 2017), and disability populations (Saunders et al., 2006), our findings with an autistic sample support a positive association between gaining employment and one’s education level. It is important to note, however, that autistic people experience high dropout rates and challenges in higher education (Flower, Richdale, & Lawson, 2021; Nuske et al., 2019), with lower rates of university and vocational education completion than the general population and other disability groups (Australian Bureau of Statistics, 2019). This might suggest that factors associated with challenges attending or completing post-secondary education might also underpin challenges gaining employment. Our findings highlight the importance of education in gaining and maintaining stable employment and present a potential avenue and venue for supporting transition age autistic people.

Beyond education level, however, none of the other available predictors distinguished the unemployed class from the reducing employment class. This might suggest that for this group, success in education may translate to early success in accessing employment, but then other factors may have impacted their ability to keep their job. With autistic adults often working in roles beneath their skills and qualifications (Hedley et al., 2023; Zwicker et al., 2017), translation of educational attainment to employment may be especially difficult for some autistic adults.

Current findings suggest that having a co-occurring condition may impact access to membership in the employed and the increasing employment classes. This was especially true for the employed class participants with a co-occurring condition reducing the odds of group membership by 57%. Although one previous autism population study from the United States found no significant association between co-occurring conditions and employment (Ohl et al., 2017), previous autism research has largely suggested that co-occurring cognitive, physical and psychological conditions negatively impact employment (for a review, see Holwerda et al., 2012). While it was beyond the scope of the present study to identify specific co-occurring conditions that might predict class membership, our findings are consistent with the notion that co-occurring conditions are an important factor contributing to stable employment. This finding has important implications for research, clinical and applied (i.e. employment programmes) practice.

Some factors uniquely explained membership in the employed class over those of who experienced consistent probability of unemployment. Later age of diagnosis was significantly positively associated with membership of the employed class. While gender has been an inconsistent predictor of employment in previous autism research (e.g. Kaya et al., 2016; Maslahati et al., 2022; Ohl et al., 2017), we found identifying as male was a positive predictor for those who reported stable employment. This difficulty is reflective of lower employment rates females experience in the general population (Bury et al., 2024). This can be accounted for by challenges often more frequently experienced by females (e.g. caring responsibilities; Hayward et al., in press), but also may reflect the additional challenges autistic women face, such as challenges due to masking, mental health, and social and communication difficulties (Hayward et al., 2018).

Higher levels of total autism traits have been found to be associated with greater difficulty in gaining employment (e.g. Howlin & Magiati, 2017; Taylor et al., 2015; Taylor & Seltzer, 2011). We found that higher levels of autism traits were associated with reduced likelihood of being in the employed class. Only one study previously has reported a positive association between autistic traits and success in employment (Harvery et al., 2021). In that small study, the authors postulated that the AQ-Short may measure aspects of autism that facilitate employment, such as preference for repetitive tasks. Nonetheless, this overlooks studies identifying significant relationships between autistic traits and factors that may be a barrier to obtaining or maintaining employment, such as mental health (Hedley et al., 2021). As we found that higher levels of autism traits were associated with lower odds of being in the employed class, more developed models of mechanisms underlying relationships between autistic traits and employment outcomes are needed. This could include acknowledging other mediating factors that impact the relationship between autism and accessing employment such as stigma (Flower, Dickens, & Hedley, 2021) or workplace design (e.g. sensory environment) and processes (Bury et al., 2021; Hedley et al., 2017; Nicholas et al., 2018).

### Limitations and future directions

While this study identifies unique trajectory of autistic people and highlights some factors that may predict membership in these profiles, these were not exhaustive. Future research should consider other factors suggested to predict employment (e.g. living situation, ability to use public transport), to better understand group membership. Another important thing to consider is the quality and meaningfulness of employment. While this study highlights the success more broadly in gaining and maintaining employment, it does not consider the potential for underemployment and employment mismatched to the autistic employee’s skills and training seen in the broader literature (Cimera & Cowan, 2009) and this sample (Bury et al., 2024). Although employment success is an important outcome for autistic adults, this should reflect meaningful employment, an area of focus for future research.

## Conclusion

Our study utilised person-oriented analyses to advance understanding of the longitudinal challenges autistic adults experience maintaining employment, and individual, family and vocational factors that may predict stability of employment over time. Future research should employ more nuanced predictors (e.g. co-occurring condition type) to help explain employment trajectory membership more precisely. However, the current findings identified several areas where supports can be directed that may facilitate improved employment outcomes for autistic adults. For example, compared to males, autistic women were underrepresented in the stable employment class, and those with co-occurring conditions were less likely to be stably employed. This indicates that employment efforts and programmes should focus funding and efforts into reducing the inequity of access for these groups.

## Supplemental Material

sj-docx-1-aut-10.1177_13623613231225798 – Supplemental material for Employment profiles of autistic people: An 8-year longitudinal study Supplemental material, sj-docx-1-aut-10.1177_13623613231225798 for Employment profiles of autistic people: An 8-year longitudinal study by Simon M Bury, Darren Hedley, Mirko Uljarević, Xia Li, Mark A Stokes and Sander Begeer in Autism

## Notes

1. Additional measures were included in the OSF but were dropped due to missing values. These include individual factors, treatment for co-occurring conditions (medication, 59.7% missing; treatment load, 59.7% missing); family factors, importance of work to parents (90.8% missing) and household income (32.9% missing); and vocational factors, job coaching/guidance (62.90% missing). See OSF for more information about how these variables were measured.
