# Supplementary material for: Employment profiles of autistic people: An 8-year longitudinal study
Source: Autism. 2024 Jan 19;28(9):2322–33. doi: 10.1177/13623613231225798 (PMC11403919; doi:10.1177/13623613231225798)
Supplement: sj-docx-1-aut-10.1177_13623613231225798 – Supplemental material for Employment profiles of autistic people: An 8-year longitudinal study [file sj-docx-1-aut-10.1177_13623613231225798.docx]

# Employment profiles of autistic people: An 8-year longitudinal study.

**Supplementary File**

## Findings for the 3 class model

Fig. S1 Probabilities of employment in the three latent classes

**Table S1**

*Baseline characteristics according to LCA-derived classes (3 CLASSES)*

|  | **1 (N=828)** | **2 (N=1168)** | **3 (N=311)** | **Overall (N=2307)** |
| --- | --- | --- | --- | --- |
| **AQ** |  |  |  |  |
| Mean (SD) | 82.1 (11.1) | 84.1 (11.6) | 81.6 (10.4) | 83.0 (11.3) |
| Missing | 65 (7.9%) | 239 (20.5%) | 23 (7.4%) | 327 (14.2%) |
| **Age** |  |  |  |  |
| Mean (SD) | 40.6 (11.1) | 38.8 (13.5) | 37.4 (12.5) | 39.3 (12.6) |
| **Gender** |  |  |  |  |
| Male | 396 (47.8%) | 444 (38.0%) | 135 (43.4%) | 975 (42.3%) |
| Female | 426 (51.4%) | 711 (60.9%) | 175 (56.3%) | 1312 (56.9%) |
| Missing | 6 (0.7%) | 13 (1.1%) | 1 (0.3%) | 20 (0.9%) |
| **Education** |  |  |  |  |
| Low | 260 (31.4%) | 528 (45.2%) | 111 (35.7%) | 899 (39.0%) |
| High | 403 (48.7%) | 304 (26.0%) | 114 (36.7%) | 821 (35.6%) |
| Missing | 165 (19.9%) | 336 (28.8%) | 86 (27.7%) | 587 (25.4%) |
| **Parent Education** |  |  |  |  |
| Low | 422 (51.0%) | 566 (48.5%) | 145 (46.6%) | 1133 (49.1%) |
| High | 314 (37.9%) | 438 (37.5%) | 138 (44.4%) | 890 (38.6%) |
| Excluded | 92 (11.1%) | 164 (14.0%) | 28 (9.0%) | 284 (12.3%) |
| **Age of Diagnosis** |  |  |  |  |
| Mean (SD) | 35.8 (12.6) | 32.5 (16.1) | 32.3 (14.4) | 33.7 (14.8) |
| Missing | 58 (7.0%) | 100 (8.6%) | 21 (6.8%) | 179 (7.8%) |
| **Co-occurring Conditions** |  |  |  |  |
| No | 473 (57.1%) | 436 (37.3%) | 150 (48.2%) | 1059 (45.9%) |
| Yes | 318 (38.4%) | 663 (56.8%) | 146 (46.9%) | 1127 (48.9%) |
| Missing | 37 (4.5%) | 69 (5.9%) | 15 (4.8%) | 121 (5.2%) |
| **Urbanicity** |  |  |  |  |
| Mean (SD) | 2.43 (1.25) | 2.64 (1.33) | 2.45 (1.29) | 2.54 (1.30) |
| Missing | 47 (5.7%) | 46 (3.9%) | 9 (2.9%) | 102 (4.4%) |

Results of the multinomial logistic regression of predictors of class membership with a 3-class model (class 2 as the reference class)

Table S2

*Results of the multinomial logistic regression of predictors of class membership with a 3-class model with the stable unemployed group as the comparison group*

| **Profile** | **1** | | | **3** | | |
| --- | --- | --- | --- | --- | --- | --- |
|  | **OR** | **95% CI** | ***p*** | **OR** | **95% CI** | ***p*** |
| **AQ** | 0.98 | 0.97, 1.00 | **0.006** | 0.99 | 0.97, 1.01 | 0.2 |
| **Age** | 0.95 | 0.92, 0.97 | **<0.001** | 0.95 | 0.92, 0.99 | **0.014** |
| **Gender** |  |  |  |  |  |  |
| Male | — | — |  | — | — |  |
| Female | 0.46 | 0.34, 0.61 | **<0.001** | 0.58 | 0.39, 0.86 | **0.006** |
| **Education** |  |  |  |  |  |  |
| Low | — | — |  | — | — |  |
| High | 2.33 | 1.78, 3.05 | **<0.001** | 1.81 | 1.26, 2.62 | **0.002** |
| **Parent Education** |  |  |  |  |  |  |
| Low | — | — |  | — | — |  |
| High | 1.16 | 0.88, 1.53 | 0.3 | 1.14 | 0.78, 1.66 | 0.5 |
| **Dx Age** | 1.04 | 1.01, 1.07 | **0.005** | 1.01 | 0.98, 1.05 | 0.4 |
| **Co-Occurring** |  |  |  |  |  |  |
| No | — | — |  | — | — |  |
| Yes | 0.41 | 0.32, 0.54 | **<0.001** | 0.56 | 0.39, 0.80 | **0.002** |
| **Urbanicity** | 0.94 | 0.85, 1.05 | 0.3 | 1.04 | 0.91, 1.20 | 0.6 |

## Findings for the 5 class model

Fig. S2 Probabilities of employment in the five latent classes

Table S3

*Baseline characteristics according to LCA-derived classes (5 CLASSES)*

|  | **1 (N=124)** | **2 (N=763)** | **3 (N=109)** | **4 (N=1144)** | **5 (N=167)** | **Overall (N=2307)** |
| --- | --- | --- | --- | --- | --- | --- |
| **AQ** |  |  |  |  |  |  |
| Mean (SD) | 81.8 (11.1) | 82.1 (11.0) | 83.0 (10.4) | 84.0 (11.7) | 81.4 (10.5) | 83.0 (11.3) |
| Missing | 6 (4.8%) | 63 (8.3%) | 9 (8.3%) | 237 (20.7%) | 12 (7.2%) | 327 (14.2%) |
| **Age** |  |  |  |  |  |  |
| Mean (SD) | 38.0 (11.7) | 40.9 (11.0) | 41.2 (12.7) | 38.7 (13.5) | 34.9 (12.1) | 39.3 (12.6) |
| **Gender** |  |  |  |  |  |  |
| Male | 43 (34.7%) | 375 (49.1%) | 47 (43.1%) | 440 (38.5%) | 70 (41.9%) | 975 (42.3%) |
| Female | 81 (65.3%) | 383 (50.2%) | 61 (56.0%) | 692 (60.5%) | 95 (56.9%) | 1312 (56.9%) |
| Excluded | 0 (0%) | 5 (0.7%) | 1 (0.9%) | 12 (1.0%) | 2 (1.2%) | 20 (0.9%) |
| **Education** |  |  |  |  |  |  |
| Low | 41 (33.1%) | 238 (31.2%) | 32 (29.4%) | 524 (45.8%) | 64 (38.3%) | 899 (39.0%) |
| High | 52 (41.9%) | 371 (48.6%) | 37 (33.9%) | 295 (25.8%) | 66 (39.5%) | 821 (35.6%) |
| Missing | 31 (25.0%) | 154 (20.2%) | 40 (36.7%) | 325 (28.4%) | 37 (22.2%) | 587 (25.4%) |
| **Parent Education** |  |  |  |  |  |  |
| Low | 52 (41.9%) | 395 (51.8%) | 53 (48.6%) | 556 (48.6%) | 77 (46.1%) | 1133 (49.1%) |
| High | 60 (48.4%) | 282 (37.0%) | 45 (41.3%) | 425 (37.2%) | 78 (46.7%) | 890 (38.6%) |
| Missing | 12 (9.7%) | 86 (11.3%) | 11 (10.1%) | 163 (14.2%) | 12 (7.2%) | 284 (12.3%) |
| **Age of Diagnosis** |  |  |  |  |  |  |
| Mean (SD) | 32.9 (13.3) | 36.2 (12.5) | 36.3 (14.5) | 32.4 (16.1) | 29.5 (14.3) | 33.7 (14.8) |
| Missing | 16 (12.9%) | 44 (5.8%) | 7 (6.4%) | 99 (8.7%) | 13 (7.8%) | 179 (7.8%) |
| **Co-occurring Conditions** |  |  |  |  |  |  |
| No | 65 (52.4%) | 438 (57.4%) | 48 (44.0%) | 430 (37.6%) | 78 (46.7%) | 1059 (45.9%) |
| Yes | 52 (41.9%) | 291 (38.1%) | 54 (49.5%) | 646 (56.5%) | 84 (50.3%) | 1127 (48.9%) |
| Missing | 7 (5.6%) | 34 (4.5%) | 7 (6.4%) | 68 (5.9%) | 5 (3.0%) | 121 (5.2%) |
| **Urbanicity** |  |  |  |  |  |  |
| Mean (SD) | 2.37 (1.30) | 2.44 (1.25) | 2.47 (1.30) | 2.64 (1.32) | 2.50 (1.30) | 2.54 (1.30) |
| Missing | 7 (5.6%) | 41 (5.4%) | 4 (3.7%) | 46 (4.0%) | 4 (2.4%) | 102 (4.4%) |

Table S4

*Results of the multinomial logistic regression of predictors of class membership with a 5-class model with the stable unemployed group as the comparison group*

| **Profile** | **1** | | | **2** | | | **3** | | | **5** | | |
| --- | --- | --- | --- | --- | --- | --- | --- | --- | --- | --- | --- | --- |
|  | **OR** | **95% CI** | ***p*** | **OR** | **95% CI** | ***p*** | **OR** | **95% CI** | ***p*** | **OR** | **95% CI** | ***p*** |
| **AQ** | 0.99 | 0.96, 1.01 | 0.4 | 0.99 | 0.97, 1.00 | **0.018** | 1.02 | 0.99, 1.05 | 0.2 | 0.98 | 0.96, 1.00 | 0.057 |
| **Age** | 0.97 | 0.91, 1.03 | 0.3 | 0.94 | 0.91, 0.97 | **<0.001** | 1.02 | 0.96, 1.08 | 0.5 | 0.93 | 0.89, 0.98 | **0.003** |
| **Gender** |  |  |  |  |  |  |  |  |  |  |  |  |
| Male | — | — |  | — | — |  | — | — |  | — | — |  |
| Female | 0.58 | 0.32, 1.05 | 0.073 | 0.44 | 0.33, 0.59 | **<0.001** | 0.58 | 0.30, 1.11 | 0.1 | 0.65 | 0.40, 1.07 | 0.09 |
| **Education** |  |  |  |  |  |  |  |  |  |  |  |  |
| Low | — | — |  | — | — |  | — | — |  | — | — |  |
| High | 2.44 | 1.37, 4.33 | **0.002** | 2.29 | 1.74, 3.01 | **<0.001** | 1.63 | 0.88, 3.03 | 0.12 | 2.41 | 1.53, 3.80 | **<0.001** |
| **Parent Education** |  |  |  |  |  |  |  |  |  |  |  |  |
| Low | — | — |  | — | — |  | — | — |  | — | — |  |
| High | 2.1 | 1.19, 3.72 | **0.011** | 1.11 | 0.84, 1.48 | 0.5 | 1.11 | 0.58, 2.12 | 0.8 | 1.03 | 0.65, 1.62 | >0.9 |
| **Dx Age** | 1.01 | 0.96, 1.06 | 0.8 | 1.04 | 1.01, 1.07 | **0.003** | 0.99 | 0.94, 1.03 | 0.6 | 1.01 | 0.98, 1.06 | 0.5 |
| **Co-Occurring** |  |  |  |  |  |  |  |  |  |  |  |  |
| No | — | — |  | — | — |  | — | — |  | — | — |  |
| Yes | 0.38 | 0.22, 0.67 | **<0.001** | 0.42 | 0.32, 0.55 | **<0.001** | 0.72 | 0.39, 1.34 | 0.3 | 0.57 | 0.37, 0.90 | **0.015** |
| **Urbanicity** | 0.97 | 0.78, 1.21 | 0.8 | 0.95 | 0.85, 1.05 | 0.3 | 0.94 | 0.74, 1.20 | 0.6 | 1.09 | 0.92, 1.29 | 0.3 |

## Findings for the 6 class model

Fig. S3 Probabilities of employment in the six latent classes

Table S5

*Baseline characteristics according to LCA-derived classes (6 CLASSES)*

|  | **1 (N=183)** | **2 (N=48)** | **3 (N=1121)** | **4 (N=790)** | **5 (N=135)** | **6 (N=30)** | **Overall (N=2307)** |
| --- | --- | --- | --- | --- | --- | --- | --- |
| **AQ** |  |  |  |  |  |  |  |
| Mean (SD) | 82.2 (11.4) | 82.0 (11.4) | 84.0 (11.7) | 82.1 (11.0) | 82.7 (10.2) | 79.7 (6.90) | 83.0 (11.3) |
| Missing | 12 (6.6%) | 1 (2.1%) | 237 (21.1%) | 64 (8.1%) | 8 (5.9%) | 5 (16.7%) | 327 (14.2%) |
| **Age** |  |  |  |  |  |  |  |
| Mean (SD) | 35.2 (11.9) | 42.6 (13.5) | 38.7 (13.5) | 40.8 (11.1) | 39.7 (12.3) | 36.0 (10.2) | 39.3 (12.6) |
| **Gender** |  |  |  |  |  |  |  |
| Male | 74 (40.4%) | 19 (39.6%) | 435 (38.8%) | 385 (48.7%) | 53 (39.3%) | 9 (30.0%) | 975 (42.3%) |
| Female | 107 (58.5%) | 29 (60.4%) | 674 (60.1%) | 400 (50.6%) | 81 (60.0%) | 21 (70.0%) | 1312 (56.9%) |
| Excluded | 2 (1.1%) | 0 (0%) | 12 (1.1%) | 5 (0.6%) | 1 (0.7%) | 0 (0%) | 20 (0.9%) |
| **Education** |  |  |  |  |  |  |  |
| Low | 73 (39.9%) | 11 (22.9%) | 514 (45.9%) | 245 (31.0%) | 43 (31.9%) | 13 (43.3%) | 899 (39.0%) |
| High | 67 (36.6%) | 25 (52.1%) | 286 (25.5%) | 384 (48.6%) | 48 (35.6%) | 11 (36.7%) | 821 (35.6%) |
| Missing | 43 (23.5%) | 12 (25.0%) | 321 (28.6%) | 161 (20.4%) | 44 (32.6%) | 6 (20.0%) | 587 (25.4%) |
| **Parent Education** |  |  |  |  |  |  |  |
| Low | 86 (47.0%) | 22 (45.8%) | 544 (48.5%) | 405 (51.3%) | 65 (48.1%) | 11 (36.7%) | 1133 (49.1%) |
| High | 79 (43.2%) | 24 (50.0%) | 416 (37.1%) | 299 (37.8%) | 59 (43.7%) | 13 (43.3%) | 890 (38.6%) |
| Missing | 18 (9.8%) | 2 (4.2%) | 161 (14.4%) | 86 (10.9%) | 11 (8.1%) | 6 (20.0%) | 284 (12.3%) |
| **Age of diagnosis** |  |  |  |  |  |  |  |
| Mean (SD) | 29.6 (14.0) | 37.8 (14.4) | 32.4 (16.1) | 36.1 (12.6) | 34.5 (14.7) | 30.9 (10.8) | 33.7 (14.8) |
| Missing | 20 (10.9%) | 2 (4.2%) | 99 (8.8%) | 47 (5.9%) | 9 (6.7%) | 2 (6.7%) | 179 (7.8%) |
| **Co-occurring Conditions** |  |  |  |  |  |  |  |
| No | 87 (47.5%) | 25 (52.1%) | 421 (37.6%) | 454 (57.5%) | 55 (40.7%) | 17 (56.7%) | 1059 (45.9%) |
| Yes | 88 (48.1%) | 22 (45.8%) | 632 (56.4%) | 301 (38.1%) | 74 (54.8%) | 10 (33.3%) | 1127 (48.9%) |
| Missing | 8 (4.4%) | 1 (2.1%) | 68 (6.1%) | 35 (4.4%) | 6 (4.4%) | 3 (10.0%) | 121 (5.2%) |
| **Urbanicity** |  |  |  |  |  |  |  |
| Mean (SD) | 2.49 (1.31) | 2.56 (1.40) | 2.64 (1.33) | 2.44 (1.25) | 2.39 (1.23) | 2.23 (1.19) | 2.54 (1.30) |
| Missing | 10 (5.5%) | 0 (0%) | 46 (4.1%) | 41 (5.2%) | 5 (3.7%) | 0 (0%) | 102 (4.4%) |

Table S6

*Results of the multinomial logistic regression of predictors of class membership with a 6-class model with the stable unemployed group as the comparison group*

| **Profile** | **1** | | | **2** | | | **4** | | | **5** | | | **6** | | |
| --- | --- | --- | --- | --- | --- | --- | --- | --- | --- | --- | --- | --- | --- | --- | --- |
|  | **OR** | **95% CI** | ***p*** | **OR** | **95% CI** | ***p*** | **OR** | **95% CI** | ***p*** | **OR** | **95% CI** | ***p*** | **OR** | **95% CI** | ***p*** |
| **AQ** | 0.99 | 0.97, 1.01 | 0.3 | 1 | 0.96, 1.03 | 0.8 | 0.98 | 0.97, 1.00 | **0.015** | 1.01 | 0.98, 1.03 | 0.7 | 0.97 | 0.93, 1.02 | 0.3 |
| **Age** | 0.94 | 0.90, 0.98 | **0.008** | 1.03 | 0.95, 1.12 | 0.5 | 0.94 | 0.91, 0.97 | **<0.001** | 0.99 | 0.94, 1.04 | 0.7 | 0.99 | 0.89, 1.10 | 0.8 |
| **Gender** |  |  |  |  |  |  |  |  |  |  |  |  |  |  |  |
| Male | — | — |  | — | — |  | — | — |  | — | — |  | — | — |  |
| Female | 0.7 | 0.42, 1.16 | 0.2 | 0.77 | 0.35, 1.70 | 0.5 | 0.45 | 0.34, 0.60 | **<0.001** | 0.74 | 0.42, 1.29 | 0.3 | 0.58 | 0.18, 1.86 | 0.4 |
| **Education** |  |  |  |  |  |  |  |  |  |  |  |  |  |  |  |
| Low | — | — |  | — | — |  | — | — |  | — | — |  | — | — |  |
| High | 2.52 | 1.58, 4.02 | **<0.001** | 3.2 | 1.40, 7.30 | **0.006** | 2.32 | 1.76, 3.06 | **<0.001** | 1.54 | 0.92, 2.60 | 0.1 | 0.95 | 0.31, 2.94 | >0.9 |
| **Parent Education** |  |  |  |  |  |  |  |  |  |  |  |  |  |  |  |
| Low | — | — |  | — | — |  | — | — |  | — | — |  | — | — |  |
| High | 1.05 | 0.66, 1.68 | 0.8 | 1.66 | 0.77, 3.58 | 0.2 | 1.15 | 0.86, 1.52 | 0.4 | 1.16 | 0.67, 1.99 | 0.6 | 3.93 | 1.13, 13.7 | **0.031** |
| **Dx Age** | 1 | 0.96, 1.04 | >0.9 | 1 | 0.94, 1.08 | 0.9 | 1.04 | 1.01, 1.07 | **0.002** | 1 | 0.96, 1.05 | 0.8 | 0.98 | 0.91, 1.07 | 0.7 |
| **Co-Occurring** |  |  |  |  |  |  |  |  |  |  |  |  |  |  |  |
| No | — | — |  | — | — |  | — | — |  | — | — |  | — | — |  |
| Yes | 0.49 | 0.31, 0.78 | **0.002** | 0.67 | 0.32, 1.42 | 0.3 | 0.41 | 0.31, 0.54 | **<0.001** | 0.73 | 0.43, 1.23 | 0.2 | 0.31 | 0.10, 0.99 | **0.048** |
| **Urbanicity** | 1.11 | 0.94, 1.33 | 0.2 | 1.14 | 0.87, 1.51 | 0.3 | 0.95 | 0.85, 1.05 | 0.3 | 0.82 | 0.67, 1.02 | 0.076 | 0.87 | 0.55, 1.38 | 0.6 |
